# Social Determinants of Health, the Chronic Care Model, and Systemic Lupus Erythematosus

**DOI:** 10.1155/2014/361792

**Published:** 2014-01-05

**Authors:** Edith M. Williams, Kasim Ortiz, Teri Browne

**Affiliations:** ^1^Institute for Partnerships to Eliminate Health Disparities, Arnold School of Public Health, University of South Carolina, 220 Stoneridge Drive, Suite 103, Columbia, SC 29210, USA; ^2^College of Social Work, University of South Carolina, Columbia, SC 29208, USA

## Abstract

Systemic lupus erythematosus (SLE) is a chronic inflammatory rheumatic disease that disproportionately affects African Americans and other minorities in the USA. Public health attention to SLE has been predominantly epidemiological. To better understand the effects of this cumulative disadvantage and ultimately improve the delivery of care, specifically in the context of SLE, we propose that more research attention to the social determinants of SLE is warranted and more transdisciplinary approaches are necessary to appropriately address identified social determinants of SLE. Further, we suggest drawing from the chronic care model (CCM) for an understanding of how community-level factors may exacerbate disparities explored within social determinant frameworks or facilitate better delivery of care for SLE patients. Grounded in social determinants of health (SDH) frameworks and the CCM, this paper presents issues relative to accessibility to suggest that more transdisciplinary research focused on the role of place could improve care for SLE patients, particularly the most vulnerable patients. It is our hope that this paper will serve as a springboard for future studies to more effectively connect social determinants of health with the chronic care model and thus more comprehensively address adverse health trajectories in SLE and other chronic conditions.

## 1. Introduction

Systemic lupus erythematosus (SLE) is a chronic inflammatory rheumatic disease that is characterized by autoantibody production and multiple organ system involvement, including a high prevalence of polyarthritis [1–4]. In the United States, over the past four decades, SLE incidence has increased and claims one of the highest mortality rates among rheumatic diseases [5, 6]. SLE incidence, prevalence, morbidity, and mortality are all much higher among minorities than whites in the United States. SLE incidence rates among African American women are 3 to 4 times higher than white women, while men have much lower rates than women in general [7], and Hispanics, Asians, and Native Americans are also more likely to develop lupus than non-Hispanic Whites [8, 9]. The overall spectrum of SLE clinical presentation is similar across different ethnic groups, but there appears to be some differences in the autoantibody profile, frequency of certain specific disease complications, and the severity and overall prognosis of the condition [10]. For example, there is cumulative evidence that lupus nephritis (LN)/renal disease is more prevalent in African and Hispanic Americans, as well as Chinese and other Asians [10–13]. It has also been shown that poverty negatively impacts disease outcomes for minority SLE patients [14–21]. Additionally, SLE patients often endure high degrees of psychological symptoms including anxiety, depression, mood disorders, and decreased health-related quality of life [12, 22–28]. These trends may be more pronounced in African and Hispanic Americans, who have been historically exposed to a unique set of risk factors that lead to a pattern of cumulative disadvantage over time [29–41].

Public health attention to SLE has been predominantly epidemiological [6], documenting mortality and morbidity of SLE and potential effects of environmental exposures [42–45]. SLE research has not traditionally embraced models around the social determinants of health and chronic care simultaneously. To better understand the effects of this cumulative disadvantage and ultimately improve the delivery of care, specifically in the context of SLE, we propose that such approaches are necessary to improve care for SLE patients, particularly for those at highest risk. Further, we suggest drawing from the chronic care model (CCM) for an understanding of how community-level factors may exacerbate disparities explored within social determinant frameworks or facilitate better delivery of care for SLE patients.

Grounded in SDH frameworks and the CCM, this paper presents issues relative to accessibility to suggest that more transdisciplinary research focused on the role of place could improve care for SLE patients, particularly the most vulnerable patients. An approach that comprehensively examines both community- and individual-level social determinants contributing to negative impacts of SLE could improve clinical knowledge (e.g., recruitment for interventions, clinical trials) and assist in improving care for those disproportionately impacted by SLE (e.g., racial and ethnic minorities).

## 2. Literature Review

### 2.1. Social Determinants of Health Framework

Originally coined by Whitehead and Dahlgren [46], the social determinants of health (SDH) framework takes into account the social, environmental, and economic conditions that impact individual health outcomes (see Table 1). This model considers the roles of both macro- and microlevel factors in population health [46–48].

Expanding on Whitehead and Dahlgren's model, Robinson's SDH framework considers the importance of community and race/ethnicity to individual health outcomes [49]. The model adheres to principles and guided activities related to surveillance, research, and program and policy development, such as (a) heterogeneity, diversity, and inclusivity; (b) a participatory approach; (c) development of trust; (d) community, race, and ethnicity; (e) community competence, development, and prevention; and (f) comprehensiveness [49]. Application of this model to SLE patients, therefore, requires activities that are aimed at not only understanding community influences but also producing activities that are participatory in addressing the needs of SLE patients.

### 2.2. Chronic Care Model

**Figure 1:**
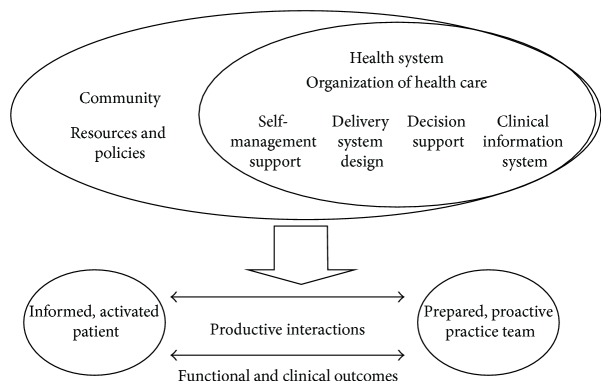
Chronic care model.

Complementing an SDH framework, Wagner's chronic care model [50, 51] (CCM) is a widely accepted model for understanding how to best organize the delivery of care to improve patient health outcomes, through six interrelated system changes that aim to make care more patient-centered and oriented around evidence-based practices (see Figure 1). The main aim of the CCM is to transform daily care for patients with chronic illnesses from acute/ambulatory treatments to more proactive approaches, with increased attention to primary care and delivery of care. Adaptation of this model results in a variety of approaches, such as effective team care and planned interactions, self-management support bolstered by more effective use of community resources, integrated decision support, and patient registries and other supportive information technology [51]. The model supports improvements in care delivery that consider community resources and maximize usage of these resources. The model has been applied in various populations and has been effective in treating and caring for the chronically ill [52–55].

One such strategy has been the implementation of self-management techniques for rheumatic, particularly SLE, patients. Over the past 25 years, research has demonstrated the effectiveness of arthritis self-management education delivered by small-group, home study, computer, and Internet modalities [36, 56–72]. Such programs have demonstrated significant improvements in health distress, self-reported global health, and activity limitation, with trends toward improvement in self-efficacy and mental stress management [41, 56, 73–77]. Consequently, numerous national agencies have recommended arthritis self-management education to complement medical care [25, 43–47]. However, a study utilizing CDSMP reported that less than 50% of a closed eligible population participated, even when Internet and small-group programs were offered repeatedly over many years [36, 62, 65, 71, 78–82]. Understanding travel impediments and other social determinants of SLE could begin to fill such gaps.

Drawing from the CCM for an understanding of how community-level factors may exacerbate disparities or facilitate better delivery of care for SLE patients within SDH frameworks is based on a paradigm shift from the current model of dealing with acute care [83] issues to a system that is prevention based [84–92]. The premise of the CCM is that quality care for the chronically ill is not delivered in isolation and can be enhanced by community resources, self management support, delivery system redesign, decision support, clinical information systems, and organizational support working in tandem to enhance patient-provider interactions (see Figure 1) [92].

### 2.3. Accessibility/Travel

Examinations of health-related travel and accessibility draw our discussion of connecting SDH frameworks and the CCM together. We reviewed scientific literature that has examined travel as a potential barrier to accessing care to assess trends, discrepancies, strengths, and limitations of current attention to social determinants impacting the lives of SLE patients.

Accessibility to care is an important component in understanding the influence of social determinants of health, and it has been addressed in health service policy research for several years [93, 94]. Considering travel, which influences patient access to healthcare, is critical to fully understand accessibility. This line of research can be extremely useful when considering the geographical availability of rheumatologists and corresponding patient needs. Research with SLE patients has suggested that economic consequences and costs of illness can negatively impact disease activity/damage and SLE patients' ability to successfully manage their disease [17, 95]. Many studies have documented racial disparities among SLE patients, but there has been little in-depth research on the social dimensions that shape these disparities, particularly with regard to travel impediments that might influence accessibility [96–108].

We were able to identify one study that specifically addressed health-related travel among SLE patients. Gillis and colleagues (2007) evaluated the association between Medicaid insurance and distance traveled by patients to treating physicians and health care utilization for SLE patients [109]. Using residential address information and primary SLE provider address information, researchers calculated the distance between the two locations for each participant, using MapQuest. They found that Medicaid patients, particularly those under the care of a rheumatologist, traveled longer distances to their primary SLE providers [109]. Medicaid patients were also more likely to be seen by a general practitioner or in the emergency room (ER) for SLE complications; travel impediments to accessing rheumatologists could have contributed to this statistic [109]. This research suggests that understanding health system infrastructure and community resources is a plausible step toward improving accessibility, potentially ensuring improved care for SLE patients, and decreasing health care costs associated with ER overuse.

In our own work, a validated psychosocial stress intervention was piloted among a cohort of African American SLE patients participating in an SLE database project at the Medical University of South Carolina (MUSC). During the course of the Balancing Lupus Experiences with Stress Strategies (BLESS) study, it became apparent that travel issues were preventing the full participation of the MUSC cohort. During followup phone calls for this project, many participants relayed that they could not participate in all aspects of the intervention because of complications related to travel. Some identified having to utilize Medicaid supported travel that required prior scheduling well in advance, but even this type of transportation was not completely reliable. Others identified having to travel long distances, which required advance planning because of reliance on family members or friends to assist with transport (unpublished observations). This information contributed to our knowledge concerning nonadherence and substantiated a need for further investigation of these issues. Specifically, this knowledge provided a foundation to investigate whether travel burden contributed to stress that may also impact the effectiveness of disease self-management programs. In that subsequent investigation, we identified four major areas of concern with respect to travel burden in accessing their rheumatologists: general travel issues; competing priorities; social/economic support challenges; and challenges surrounding general health (unpublished observations).

All of these findings emphasize the importance of exploring the specific factors that limit and motivate the participation of a vulnerable disease population in critical healthcare and research activities.

## 3. Conclusion

In conclusion, this review of SDH frameworks, the CCM, and the case of accessibility and travel issues in the context of SLE reveal that much more work can be done to improve care for SLE patients. Transdisciplinary approaches and research efforts could bring together several innovative methods for better addressing the complex issues faced by SLE patients. Further investigation of exactly how current approaches have been limited and the implications for disease manifestations and treatment are warranted to guide future SLE research. This would help to assess the appropriateness of focusing more broadly on “upstream” influences as a way to improve quality of life for SLE patients by exploring instances where focusing on upstream influences has improved care outcomes. Thus, we would more comprehensively elucidate the need for population health approaches rooted in the social determinants of health framework while setting the stage for connecting it to a biomedical framework that is widely accepted among physicians and medical researchers. By this, we can suggest not only continuing traditional models of biomedical research but also producing extrapolations regarding how broad social influences impact the lives of SLE patients.

Our review suggests that more research is needed to expand the scope of barriers of SLE to include social determinants such as accessibility and health-related travel. GIS research methods should be used in future research to begin to fill this knowledge gap. Thus, drawing from geography research methods and theories, researchers attempting to better understand place-related disparities could benefit greatly from transdisciplinary intervention activities that include research areas that explore the impact of travel on SLE patients, especially considering the geographic coverage of clinical trials studying SLE. It is our hope that this paper will serve as a springboard for future studies to more effectively connect social determinants of health with the chronic care model and thus more comprehensively address adverse health trajectories in SLE and other chronic conditions.

## Figures and Tables

**Table 1:** Whitehead and Dahlgren's social determinants of health framework components.

Components	Examples
(1) General socioeconomic, cultural, and environmental conditions	Proximity to industrialized, toxic facilities
(2) Living and working conditions	Agriculture and food production; education; work environment; unemployment; water and sanitation; health care services; housing
(3) Social and community networks	Social capital networks; civic organizations
(4) Individual lifestyle factors	Smoking behavior; sexual risk factors
(5) Age, sex, and constitutional factors	Biological determinants
